# Brazilian Dialysis Survey 2024

**DOI:** 10.1590/2175-8239-JBN-2025-0112en

**Published:** 2026-02-16

**Authors:** Fabiana Baggio Nerbass, Helbert do Nascimento Lima, Bruno Zawadzki, José A. Moura-Neto, Jocemir Ronaldo Lugon, Ricardo Sesso

**Affiliations:** 1 Fundação Pró-Rim, Joinville, SC, Brazil.; 2 Universidade da Região de Joinville, Joinville, SC, Brazil.; 3 Davita Tratamento Renal, Rio de Janeiro, RJ, Brazil.; 4 Escola Bahiana de Medicina e Saúde Pública, Salvador, BA, Brazil.; 5 Universidade Federal Fluminense, Niterói, RJ, Brazil.; 6 Universidade Federal de São Paulo, São Paulo, SP, Brazil.

**Keywords:** Renal Dialysis, Peritoneal Dialysis, Hemodiafiltration, Epidemiology, Renal Insufficiency, Chronic

## Abstract

**Introduction:**

The annual Brazilian Dialysis Survey (BDS) plays an important role in informing and shaping national health policies.

**Objective:**

To present the 2024 epidemiological findings from the BDS conducted by the Brazilian Society of Nephrology (BSN) and compare them with previous years.

**Methods:**

A survey was conducted among Brazilian chronic dialysis centers through voluntary participation, utilizing an online questionnaire to assess clinical and epidemiological characteristics of dialysis patients, as well as dialysis center attributes. For specific estimates of prevalence, incidence, and funding source, a nationally representative random sample of dialysis centers stratified by geographic region was selected (n = 258).

**Results:**

A total of 386 dialysis centers (42.7%) voluntarily responded to the online questionnaire, and 162 centers from the randomly selected centers provided data. On July 1st, 2024, the estimated number of dialysis patients was 172,585, with 52,944 new patients starting dialysis in 2024. The estimated prevalence and incidence rates per million population (pmp) were 812 and 249, respectively. Among prevalent patients, 87.3% were undergoing hemodialysis, 7.1% hemodiafiltration, and 5.6% peritoneal dialysis. Compared to the previous year, there was an increase in catheter use for hemodialysis vascular access, along with higher prevalence rates of anemia, hyperphosphatemia, hyper­kalemia, and low Kt/V. The estimated crude annual mortality rate was 16.5%.

**Conclusions:**

Data from a random sample of dialysis centers indicate a continued rise in the number and prevalence of chronic dialysis patients in Brazil. Worsening trends in permanent vascular access, dialysis adequacy, and metabolic control underscore the need for targeted improvements in patient care.

## INTRODUCTION

With an estimated population of 212.6 million in July, 2024, Brazil is currently the 7th most populous country in the world^1^, with a prevalence of chronic dialysis that is approximately twice as high as global estimates^2^.

The cost of chronic kidney replacement therapy by dialysis has been covered by the Brazilian government through the Unified Health System (SUS), for most Brazilians, for over 30 years. Although reimbursement rates for dialysis therapy are low compared to those in other countries, treatment prevalence rates in Brazil are comparable to those of many developed nations. The Brazilian Society of Nephrology (BSN) has been continuously advocating for fair reimbursement from the government. More recently, international companies have expanded their presence in the country as dialysis providers, contributing to a greater diversity of treatment options and improved standardization of procedures.

For more than two decades, the BSN has conducted the Brazilian Dialysis Survey (BDS), a nationwide online survey designed to collect and analyze epidemiological and clinical data on patients undergoing chronic dialysis. This initiative has been instrumental in generating valuable insights to inform health policies and improve the care of individuals receiving chronic dialysis treatment in Brazil.

As previously performed in the 2023 BDS^3^, in this report, data were collected both from a voluntary survey and from a randomly selected sample of dialysis facilities across the country. This approach aims to enhance the accuracy of prevalence and incidence rates, as well as payment source estimates.

Here, we aimed to present the main findings of the 2024 BDS and compare them with previous survey results.

## METHODS

This was a cross-sectional study, with inclusion criteria defined as dialysis facilities having an active registration with the BSN.

### Prevalence, Incidence, and Source of Payment for Dialysis

For estimates of dialysis prevalence, incidence, and source of payment, data were obtained through random sampling from a list of dialysis centers in each geographic region (North, Northeast, Central-West, Southeast, and South). The centers were selected through a computer-based random sampling method. Approximately 30% of the dialysis centers in each geographic region were selected (n = 258). A minimum target of 60% (n = 155) of the planned number of dialysis facilities was established. These proportions were set based on calculations performed for the 2023 survey, which yielded acceptable estimates and 95% confidence intervals.

The selected dialysis units were first contacted via e-mail asking the following data: 1. Total number of patients on chronic dialysis on July 1^st^, 2024; 2. Number of patients with private healthcare insurance for dialysis funding on July 1^st^, 2024; and 3. Number of patients who started chronic dialysis during July 2024. To increase the response rate, the units that did not answer the electronic messages were contacted by telephone.

To calculate the estimates, the country’s regions (North, Northeast, Central-West, Southeast, and South) were considered as strata. As a result, weights were assigned to regions in analysis stage to compensate for slight variations in sampling probabilities and maintain proportionality (Table 1). Considering that response rates were somewhat different by region, weights were assigned to allow the distribution of the sample across the strata to be the same as that observed in the study population. Given the sampling method employed (simple stratified sampling), the average dialysis patients per center was estimated on July 1^st^, and the average (95% CI) number of new patients in the year for each region was calculated. The results were then expanded to the total number of units in each region. The calculations presented in Table 1 were made in the Stata software using the ‘svy’ module, which allows considering the weights of the sample elements, the correction for the finite population, and the stratification. The percentages of patients financed by public and private healthcare insurance were obtained through simple proportion in the drawn sample.

**Table 1:** Sampling procedures in dialysis facilities across the country, sampling fractions, design weights, response rates, and adjusted weights by region

Region	Facilities			Sampling fraction	Design weight	Response rate	Adjusted weight
	Existing	Sampled	Responding				
North	58	19	14	0.328	3.053	0.737	4.14286
Northeast	172	48	30	0.279	3.583	0.625	5.73333
Central-West	91	30	17	0.330	3.033	0.567	5.35294
Southeast	425	118	72	0.278	3.602	0.610	5.90278
South	158	43	29	0.272	3.674	0.674	5.44828
Total	904	258	162			0.628	

Notes – Sampling fraction = sampled/existing centers; Design weight = 1/sampling fraction; Response rate = responding/sampled centers; Adjusted weigh t = design weight/response rate.

### Data from the Voluntary Survey

Individuals designated by the participating dialysis centers completed an online questionnaire available on the BSN website, use to collect data on the sociodemographic, clinical, and therapeutic characteristics of patients undergoing chronic dialysis. The questionnaire was accessible from August to November 2024. Participation was voluntary, and all dialysis centers registered with BSN were invited via email and BSN media channels. To maximize participation, monthly reminders were sent to centers that had not yet submitted data, and direct contact was established with BSN regional presidents and managers of international dialysis service providers in Brazil.

Each center provided aggregate data, rather than individual patient information, on the prevalence of predefined characteristics (e.g., the number of patients on hemodialysis, those with diabetic kidney disease, male patients, etc.). Annual mortality was estimated based on occurrences from July 2024. To calculate prevalence and incidence rates, we used national and regional 2024 population data from the Brazilian Institute of Geography and Statistics (IBGE): 212.6 million inhabitants^1^.

Most data were descriptive and compared with data from previous years. Estimated total annual number of deaths was obtained from the number of deaths reported in July multiplied by 12, and divided by the proportion of active participating centers. Estimated crude annual mortality rate (%) was obtained from the estimated total number of deaths in 2024 multiplied by 100 and divided by the estimated number of dialysis patients on July 1^st^ in the voluntary sample.

This study did not involve access to primary or secondary individual patient data. All information was obtained in aggregate form, as reported by the participating dialysis centers through a standardized online questionnaire.

## RESULTS

In July 2024, a total of 904 active chronic dialysis centers were registered with BSN, representing a 2% increase from 2023. Nationwide, there were 4.2 dialysis centers per million population (pmp). Regional disparities were observed, with lower rates in the Northeast (3.0 pmp) and North (3.1 pmp) compared to higher rates in the Southeast (4.8 pmp), Central-West (5.3 pmp), and South (5.1 pmp) regions.

### Random Sample Estimates

#### Estimated Incidence and Prevalence Rates from the Random Sample

Eighteen percent of the active Brazilian dialysis centers participated in the random sample (n = 162/904), 24% of the North (n = 14/58), 17% of the Northeast (n = 30/172), 19% of the Central-West (n = 17/91), 17% of the Southeast (n = 72/425) and 18% of the South (n = 29/158) centers. The centers represented 62% of the planned total (162 out of 258).

**Figure 1.**
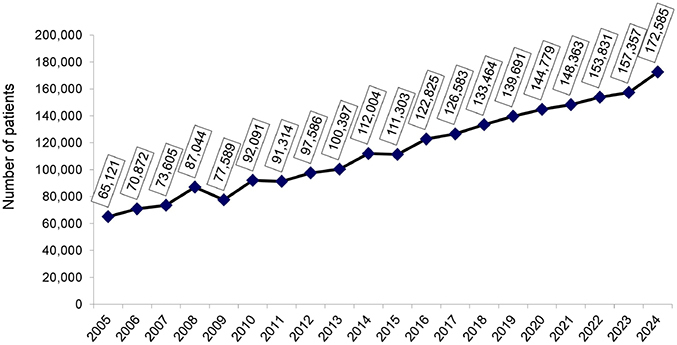
Estimated number of patients on chronic dialysis per year.

**Figure 2.**
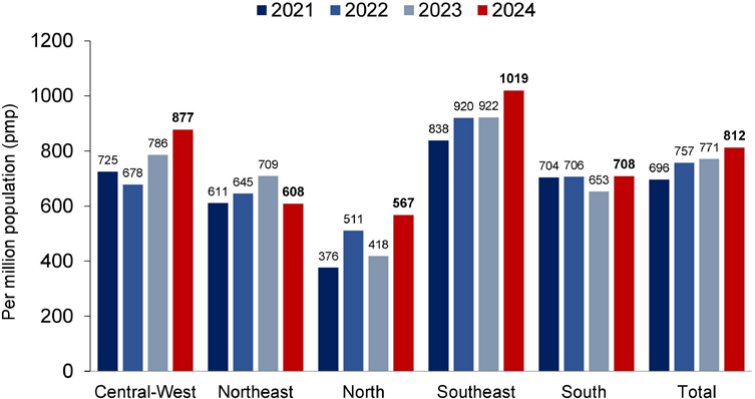
Estimated prevalence rate of patients on dialysis by geographic region in Brazil, per million population.

On July 1, 2024, the estimated total number of dialysis patients was 172,585 (95% CI: 156,687–188,483), reflecting a 9.7% increase from July 2023—the largest annual increase observed in recent years (Figure 1). The prevalence of dialysis patients also continued to rise, from 771 pmp in 2023 to 812 pmp (95% CI: 737–887 pmp) in 2024. Regionally, prevalence rates declined in the Northeast (Figure 2).

The estimated number of new dialysis patients in 2024 was 52,944, with an overall incidence rate of 249 pmp (95% CI: 220–279 pmp), comparable to the 2023 rate of 251 pmp. Incidence rates varied across regions, ranging from 120 pmp in the North to 311 pmp in the Southeast.

Based on the random sample survey, 79% of dialysis patients were funded by the public health system, while 21% relied on private health insurance. Public funding varied by region, being highest in the North (91%), followed by the Northeast (80%), Southeast (78%), South (76%), and Central-West (70%).

### Voluntary Survey

A total of 386 dialysis centers (43%) participated in the voluntary survey, the highest response rate recorded since 2014. When analyzing participation by region, the Northeast had the highest response rate (49%), followed by the South (46%), Central-West (42%), Southeast (40%), and North (34%).

**Figure 3.**
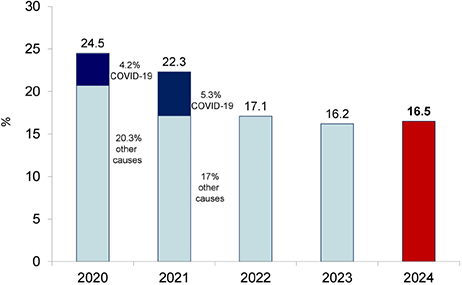
Estimated annual crude mortality rate of dialysis patients.

The number of patients included in the current BDS was 22.9% higher than in 2023, increasing from 62,617 to 76,946. The estimated number of deaths for the year was 29,712, resulting in an annual crude mortality rate of 16.5% (Figure 3).

#### Demographic and Clinical Characteristics

Fifty nine percent of dialysis patients were men and 41% were women. In terms of age distribution, 0.8% were younger than 20 years, 61.4% were between 20 and 64 years, and 37.8% were aged 65 or older.

**Figure 4.**
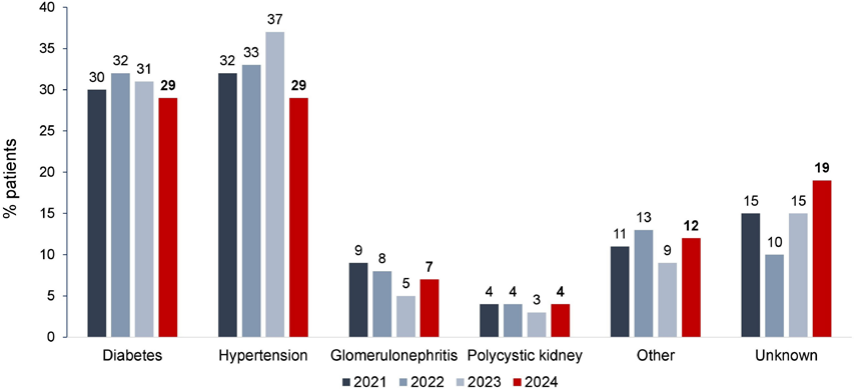
Distribution of dialysis patients according to chronic kidney disease etiology.

**Figure 5.**
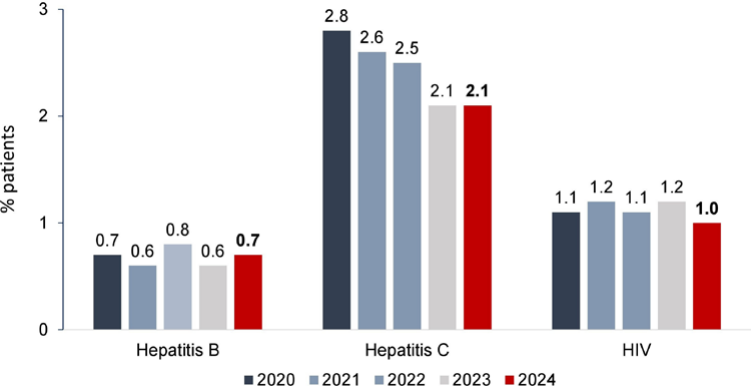
Prevalence of patients with positive serology for hepatitis B and C and HIV.

Hypertension and diabetes remained the leading underlying causes of kidney failure, each accounting for 29% of cases (Figure 4). The prevalence of patients with positive serology for hepatitis C, hepatitis B, and HIV remained stable and similar to that of 2023 (Figure 5).

**Figure 6.**
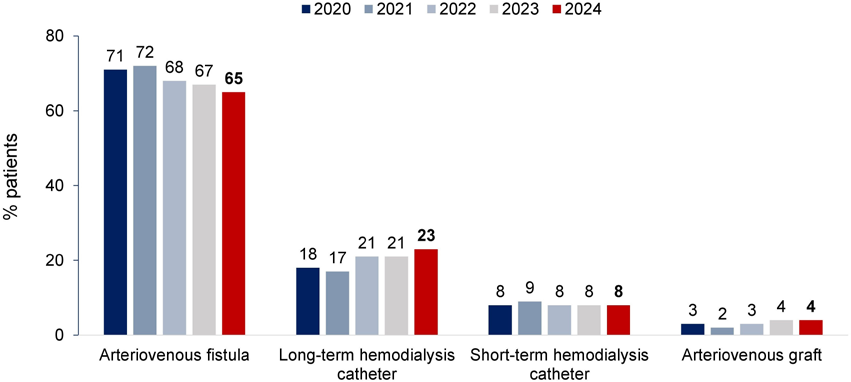
Type of vascular accesses used for hemodialysis.

Regarding vascular access for hemodialysis or hemodiafiltration, nearly a quarter of patients (23%) used a long-term catheter, while the prevalence of arteriovenous fistula decreased (Figure 6).

**Figure 7.**
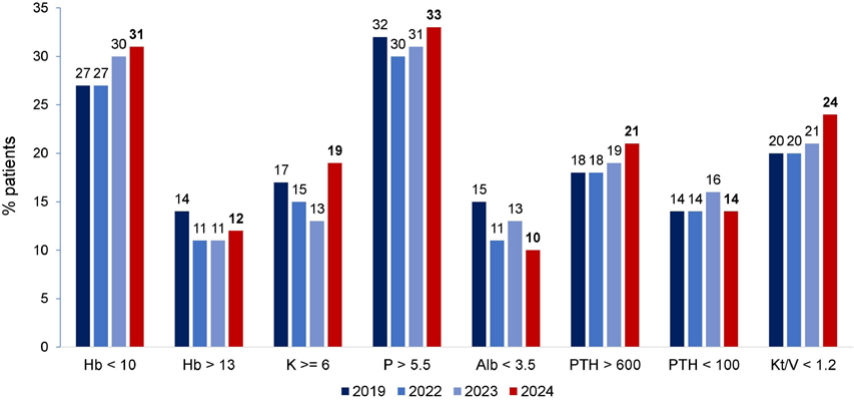
Distribution of dialysis patients according to biochemical results.

Most biochemical parameters worsened compared to the previous year, particularly the prevalence of serum potassium ≥ 6.0 mEq/L, which increased from 13% to 19%, and inadequate Kt/V, which rose from 21% to 24%. Additionally, 21% of patients had paratormone >600 pg/mL, 31% of patients had hemoglobin levels <10 g/dL, and one-third of the population had serum phosphate >5.5 mg/dL (Figure 7).

**Figure 8.**
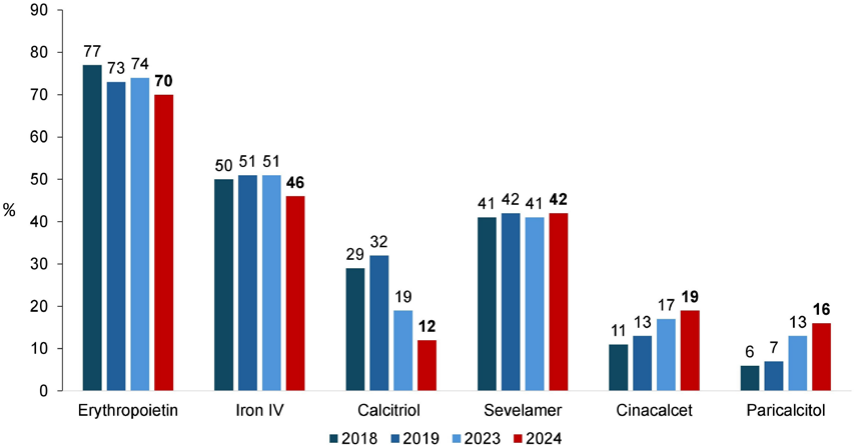
Distribution of dialysis patients according to the use of medications.

The main changes in medication use compared to the previous year were a decrease in erythropoietin use (74% to 70%), intravenous iron (51% to 46%), and calcitriol (19% to 12%) (Figure 8).

#### Characteristics of Dialysis Treatment

The distribution of dialysis modalities and funding sources in the voluntary survey is shown in Table 2. The public health system funded 80.7% of patients (n = 62,084/76,946), while private health insurance covered 19.3% (n = 14,862/76,946).

**Table 2:** Distribution of patients by modality of dialysis and paying source

Modality	Public health		Private health		Total	
	N	%	N	%	N	%
HD ≤ 4 sessions/week	58734	94.6	8006	53.6	66740	86.7
HD > 4 sessions/week	0	0	411	2.8	411	0.5
Home HD	0	0	29	0.2	29	0.0
HDF ≤ 4 sessions/week	201	0.3	4560	30.7	4761	6.2
HDF > 4 sessions/week	0	0	663	4.5	663	0.9
Home HDF	0	0	5	0	5	0
CAPD	308	0.5	127	0.9	435	0.6
APD	2771	4.5	1029	6.9	3800	4.9
IPD	70	0.1	32	0.2	102	0.1
Total	62084	100	14862	100	76946	100

Abbreviations – HD: hemodialysis; HDF: hemodiafiltration; CAPD: continuous ambulatory peritoneal dialysis; APD: automated peritoneal dialysis; IPD: intermittent peritoneal dialysis.

**Figure 9.**
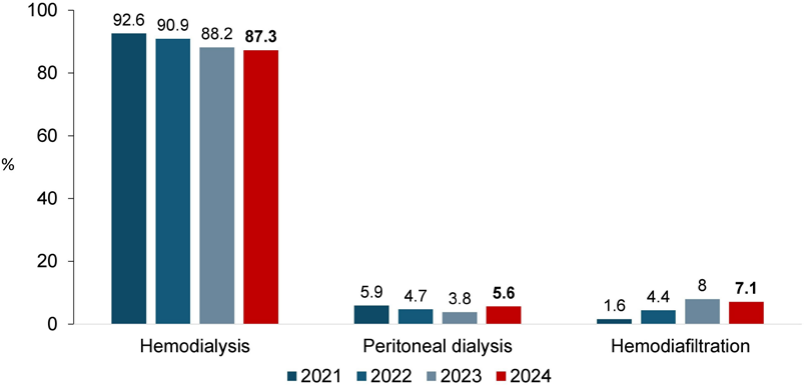
Distribution of dialysis patients according to dialysis modality.

The proportion of patients undergoing hemodialysis and hemodiafiltration showed a slight decline, decreasing from 88.2% to 87.3% and from 8.0% to 7.1%, respectively. In contrast, the use of peritoneal dialysis increased nationally, from 3.8% to 5.6% (Figure 9) and across all regions, except the North.

**Figure 10.**
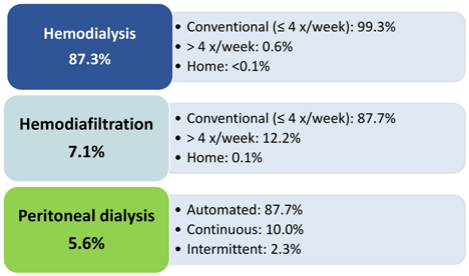
Distribution of patients according to dialysis prescription.

More than 10% of patients on hemodiafiltration had at least five sessions per week, while only a minority of peritoneal dialysis patients were not using an automated modality (Figure 10).

Regarding dialysate characteristics, 60% of dialysis facilities used the same bicarbonate concentration for all patients, and 62% used the same potassium concentration.

#### Characteristics of Participating Centers

Among the 386 participating dialysis centers, 76% were privately owned, 15% were philanthropic, and 9% were public. Additionally, 27% of the centers were operated by international corporations.

Most centers were classified as satellite units (68%), while 32% were in-hospital centers. The national average number of patients per nephrologist, registered nurse, and nurse technician was 25, 41, and 7, respectively.

## DISCUSSION

In this study, we present the main findings from the 2024 edition of the BDS. This year marked the highest participation rate in the past decade, reflecting increased engagement to the survey. Notably, the most significant trend observed was an increase in the prevalence of inadequate biochemical parameters, underscoring ongoing challenges in achieving treatment adequacy and effective metabolic control.

We presume that the marked increase in the estimated number of dialysis patients of almost 10% in one year may primarily reflect two factors: the growing elderly population in Brazil due to improved life expectancy^4^ and the lower mortality rates observed after the COVID-19 pandemic years. However, the accuracy of this estimate, based on sample data, needs to be confirmed in future surveys.

The prevalence of dialysis increased from 771 to 812 patients pmp, while regional disparities persisted, probably reflecting inequalities in treatment access across the country. On average, the prevalence rate remained 35% higher in the three most developed regions – Central-West (887 pmp), Southeast (1,019 pmp), and South (708 pmp)—compared to the less developed North (567 pmp) and Northeast (608 pmp), with a mean of 902 pmp versus 587 pmp, respectively. These discrepancies underscore the influence of socioeconomic and infrastructure factors on access to renal replacement therapy. A recent survey covering data from 167 countries reported substantial variation in chronic dialysis prevalence, with a median of 322.7 pmp for hemodialysis [interquartile range (IQR) 76.3–648.^8^]^2^ and 21.0 pmp for peritoneal dialysis [IQR 1.5–62.^4^]^5^, further contextualizing Brazil’s position as a country with relatively high dialysis coverage – comparable to several developed nations – yet marked by significant internal imbalances.

An even greater disparity is observed in the incidence rate, which is 42% higher in the most developed regions (273 pmp) than in the least developed regions (156 pmp). This highlights persistent inequities not only in ongoing treatment but also the initiation of dialysis. Nationally, the incidence rate remained relatively stable, with a slight decrease from 251 to 249 pmp compared to the previous year.

Interestingly, for the first time since the first BDS, diabetes—traditionally the second leading cause—matched hypertension as the primary cause of kidney failure, jointly occupying the top position. Concomitantly, there was a rise in the prevalence of other and unknown primary causes, which together accounted for nearly one-third of cases (31%). In other international registries, such as the USRDS from the United States and the European ERA Registry, diabetes has consistently been the leading cause of kidney failure over the past decade^6,7^. The recent convergence of diabetes and hypertension as primary causes in our data reflects a similar trend and reinforces the need for focused preventive strategies targeting diabetes-related kidney disease.

Regarding positive serology, the slight fluctuations observed in recent years for HIV and hepatitis B persisted. Notably, hepatitis C prevalence did not decrease for the first time since 1999, maintaining the 2.1% prevalence reported in 2023. Continued vigilance and reinforcement of screening and infection control practices remain critical in dialysis populations.

The use of arteriovenous fistulas for hemodialysis access continued to decline, while long-term catheters now serve as the vascular access for nearly a quarter of patients. This trend may, in part, reflect the increasing expertise of nephrologists in placing long-term catheters, increasing their availability and perceived convenience particularly if compared to non-tunneled catheters. However, it also raises concerns about the potential compromise of long-term outcomes, given the lower patency and the higher risks of infection, hospitalization, and mortality associated with catheter use compared to arteriovenous fistulas^8,9^. These findings underscore the need to balance convenience with adherence to best practices in vascular access planning, particularly timely referral for surgical evaluation and patient education.

Compared to 2023, the prevalence of inadequate biochemical parameters increased across nearly all indicators, highlighting potential challenges in treatment adequacy and metabolic control. The proportion of patients with Kt/V < 1.2 increased from 21% to 24%, suggesting a decline in dialysis dose delivery or adherence. Similarly, the rate of hyperkalemia rose from 13 to 19% and of high parathormone from 19 to 21%. Meanwhile, rates of hyperphosphatemia remained persistently high, affecting approximately one-third of patients, indicating ongoing difficulties in achieving phosphorus control despite therapeutic options. Recently, in a national survey with 114 dialysis centers, approximately 30% reported difficulties in prescribing at least 12 hours of hemodialysis per week^10^, which may partly explain these findings. The same survey also found that nearly 20% of patients present with severe hyperparathyroidism, and that many services have limited access to both clinical and surgical treatment for this condition^10^.

The prevalence of anemia remained high, affecting 31% of patients, while the use of the main treatments – erythropoietin and intravenous iron – fell to their lowest levels since data collection for these variables began in 2008, at 70 and 46%, respectively. The decline in therapeutic use, despite persistent anemia rates, is concerning and the reasons behind these trends warrant further investigation, particularly given the well-established association between untreated anemia and poorer outcomes in dialysis patients^11^.

As observed in the previous year, there was a continued decline in the use of calcitriol, alongside an increase in the use of cinacalcet and paricalcitol. This shift likely reflects the evolving clinical strategies aimed at managing secondary hyperparathyroidism more effectively while minimizing the risk of hypercalcemia and vascular calcification^12^. In addition, external factors may have contributed to these trends, such as the market discontinuation of intravenous calcitriol. A previous national survey also found that a significant proportion of dialysis units reported difficulties in obtaining medications related to CKD-MBD, which may further influence treatment patterns^10^.

The use of peritoneal dialysis increased from 3.8 to 5.6%, reversing the decline observed over the past two years. In contrast, the use of hemodiafiltration decreased modestly from 8 to 7%, and that of hemodialysis from 88 to 87%. Possible explanations for the rebound in peritoneal dialysis include the higher participation rate in the present survey and the impact of public policies in several states aimed at promoting this modality.

Among study limitations, we note the reliance on electronic data collection through voluntary participation, the aggregation of patient-level data at the dialysis center level, and the lack of validation for responses. Additionally, since the incidence and prevalence rates were calculated using different sampling methods compared to previous years (before 2023), comparisons over time should be interpreted with caution. As a strength, the use of a random sampling method to select dialysis centers across our large country, home to more than 900 active facilities, enhanced the validity of prevalence and incidence estimates, as well as the accuracy of dialysis funding type distributions.

In conclusion, the 2024 BDS survey confirmed the continued rise in the prevalence of dialysis patients in Brazil, along with concerning trends in vascular access, including a decrease in arteriovenous fistula use. Additionally, the high proportion of patients with inadequate dialysis doses and poorer metabolic control highlights the need for targeted interventions to improve dialysis care and patient outcomes.

## Data Availability

The data that support the findings of this study are available from the Brazilian Society of Nephrology (SBN), but restrictions apply. Data are not publicly available due to privacy and ethical considerations, but may be obtained from the authors upon reasonable request and with permission from the SBN.

## Supplementary Material

The following online material is available for this article:

Questionnaire BDS 2024.
