# Supplementary material for: Brazilian Dialysis Survey 2024
Source: J Bras Nefrol. 2026 Feb 16;48(1):e20250112. doi: 10.1590/2175-8239-JBN-2025-0112en (PMC12922704; doi:10.1590/2175-8239-JBN-2025-0112en)
Supplement: Supplementary file 1 [file 2175-8239-jbn-48-1-e20250112-suppl.pdf]

Braz. J. Nephrol.
https://doi.org/10.1590/2175-8239-JBN-2025-0112en

Supplementary Material to “Brazilian Dialysis Survey 2024”

ATENÇÃO:

O preenchimento deverá ser obrigatoriamente feito no site e formulários enviados por e-mail ou fax não serão considerados.

No final da página clique no botão verde para "Enviar os dados", será exibida a mensagem "Enviado com sucesso!", caso contrário não foi enviado.

Após enviado é possível efetuar alterações e enviar novamente.

Após o envio com sucesso, aparecerá na linha abaixo e no final do formulário um segundo botão para imprimir o certificado de participação.

Também no final da página é possível clicar no botão "Salvar os dados mesmo que não tenha preenchido todos os campos" e posteriormente enviar todos os dados.

Questões com * são de preenchimento obrigatório.

Dados cadastrais

CNPJ:

Razão social:*

Hospital (repita a razão social caso a sua unidade não seja um hospital):

Endereço:*

Bairro:

Cidade:*

UF:* Selecione

CEP:*

Telefone:* Ramal:

Fax: Ramal:

E-mail (separe com ";" ponto e virgula, se tiver mais de um):*

Atenção: e-mails do tipo: contato@, gerencia@, recepcao@, diretoria@, etc. Podem ser rejeitados pelo nosso sistema de e-mail marketing, nos avise se for o caso.

Whatsapp com DDD:

Whatsapp de: ○ Pessoa física ○ Pessoa Jurídica

OBS: caso o telefone seja de pessoa física, solicitamos que seja preenchido, assinado e devolvido (digitalizado PDF / JPG / FOTO) o termo de consentimento a ser dado pelo TITULAR DO NÚMERO para registro@sbn.org.br.

Responsável técnico - Dr.(a):*

Responsável pelo preenchimento:*

Função:* Selecione

Perfil da unidade

Tipo: ○ Pública ○ Filantrópica ○ Privada*

Proprietários do serviço na junta comercial:

○ Corporação internacional
○ Grupo constituído por médicos nefrologistas
○ O próprio hospital onde se situa a clínica
○ Grupo constituído por médicos (nefrologista ou não) e/ou outros
○ Profissionais não médicos

Universitária? ○ Sim ○ Não

Localização: ○ Hospitalar ○ Satélite

Número de profissionais que trabalham no centro de diálise:

Nefrologistas:

Enfermeiros:

Técnicos de enfermagem:

Convênios: ○ SUS ○ Outros Comvênios ○ Ambos

Oferece serviço de diálise peritoneal? ○ Sim ○ Não

Número total de pacientes em programa crônico de diálise em 1/julho/2024:*

Número de pacientes em programa crônico de diálise no dia 1/julho/2024 por modalidade e convênio:*

	SUS	Não SUS	TOTAL
HEMODIÁLISE convencional no centro (4 x ou menos por semana)			
HEMODIÁLISE diária no centro (mais que 4 x por semana)			
HEMODIÁLISE domiciliar			
HEMODIAFILTRAÇÃO no centro (4 x ou menos por semana)			
HEMODIAFILTRAÇÃO diária no centro (mais que 4 x por semana)			
HEMODIAFILTRAÇÃO domiciliar			
CAPD			
DPA			
DPI			
TOTAL			

Banho de diálise:

Todos os pacientes em hemodiálise na clínica são tratados com a mesma concentração de **bicarbonato no banho?** ○ Sim ○ Não

Todos os pacientes em hemodiálise na clínica são tratados com a mesma concentração de **potássio no banho?** ○ Sim ○ Não

Número de pacientes novos:

(Que iniciaram diálise no mês de julho de 2024 – NÃO INCLUIR pacientes que vieram transferidos de outro programa de diálise regular, apenas os que de fato iniciaram programa crônico de diálise e não faziam diálise anteriormente).

Total:

Número de pacientes novos com diagnóstico de diabetes:

Óbitos:

Número de óbitos no mês de julho de 2024: (Se não houver, colocar 0)

Acesso para hemodiálise em 1/jul/2024:

Número de pacientes em HD usando cateter venoso central de curta permanência:

Número de pacientes em HD usando cateter venoso central de longa permanência:

Número de pacientes em HD usando enxerto com material sintético (prótese):

Infecções virais:

Número de pacientes com:

Hepatite B: Hepatite C: HIV:

Perfil dos pacientes (informar o número de pacientes)

Faixa Etária

0 a 12 anos: 45 a 64 anos:
13 a 19 anos: 65 a 74 anos:
20 a 44 anos: 75 anos ou mais:

Sexo

Masculino: Feminino:

Faixas de índice de massa corpórea (IMC Kg/m²):

Baixo peso (<18,5): Obesidade (30 - 39,9):
Normal (18,5 - 24,9): Obesidade Mórbida (≥40):
Sobrepeso (25 - 29,9):

Diagnóstico de base: (informar o número de pacientes considerando apenas um diagnóstico por paciente)

Diabetes: Rins policísticos:
Hipertensão: Outro:
Glomerulonefrite: Indefinido:

Exames laboratoriais:

Os exames laboratoriais de rotina são coletados antes da: ○ primeira sessão de diálise da semana ○ segunda sessão de diálise da semana

Resultados referentes ao mês de julho de 2024 ou referentes ao mês mais próximo em relação a julho de 2024.
Número de pacientes com:

Hemoglobina maior que 13 g/dl:

Hemoglobina menor que 10 g/dl:

Potássio maior ou igual a 6,0 mEq/l:

Fósforo maior que 5,5 mg/dl:

Albumina menor que 3,5 g/dl:

PTH menor que 100 pg/ml:

PTH maior que 600 pg/ml:

Kt/V (em HD) menor que 1,2 (ou taxa de redução de ureia menor que 65%):

Medicações em uso em 1/jul/2024 (número de pacientes):

Eritropoietina: Ferro Venoso:
Calcitriol oral: Calcitriol intravenoso:
Colecalciferol oral: Sevelamer:
Carbonato de cálcio/acetato de cálcio: Cinacalcete (Mimpara®):
Paricalcitol (Zemplar®) - não é calcitriol:

SALVAR PARA COMPLETAR DEPOIS

ENVIAR OS DADOS

Será possível enviar novamente!
