# Supplementary material for: Brazilian Dialysis Survey 2024
Source: J Bras Nefrol. 2026 Feb 16;48(1):e20250112. doi: 10.1590/2175-8239-JBN-2025-0112en (PMC12922704; doi:10.1590/2175-8239-JBN-2025-0112en)
Supplement: Translation

# Translation: Censo Brasileiro de Diálise 2024

**DOI:** 10.1590/2175-8239-JBN-2025-0112pt

**Authors:** Fabiana Baggio Nerbass, Helbert do Nascimento Lima, Bruno Zawadzki, José A. Moura-Neto, Jocemir Ronaldo Lugon, Ricardo Sesso

**Affiliations:** 1 Fundação Pró-Rim, Joinville, SC, Brazil.; 2 Universidade da Região de Joinville, Joinville, SC, Brazil.; 3 Davita Tratamento Renal, Rio de Janeiro, RJ, Brazil.; 4 Escola Bahiana de Medicina e Saúde Pública, Salvador, BA, Brazil.; 5 Universidade Federal Fluminense, Niterói, RJ, Brazil.; 6 Universidade Federal de São Paulo, São Paulo, SP, Brazil.

**Keywords:** Diálise Renal, Diálise Peritoneal, Hemodiafiltração, Epidemio­logia, Insuficiência Renal Crônica

## Abstract

**Introdução:**

O Censo Brasileiro de Diálise (CBD) anual desempenha papel fundamental na informação e definição de políticas nacionais de saúde.

**Métodos:**

Realizou-se pesquisa entre centros brasileiros de diálise crônica por participação voluntária, utilizando questionário online para avaliar características clínicas e epidemiológicas de pacientes em diálise e atributos dos centros. Para estimar a prevalência, a incidência e a fonte de financiamento, foi selecionada uma amostra aleatória nacionalmente representativa de centros de diálise, estratificada por região geográfica. (n = 258).

**Resultados:**

Um total de 386 centros de diálise (42,7%) respondeu voluntariamente ao questionário online e 162 centros dentre os selecionados aleatori­amente forneceram dados. Em 1º de julho de 2024, o número estimado de pacientes em diálise era 172.585, com 52.944 novos pacientes iniciando diálise em 2024. Taxas estimadas de prevalência e incidência por milhão da população (pmp) foram 812 e 249, respectivamente. Entre pacientes prevalentes, 87,3% estavam em hemodiálise, 7,1% em hemodiafiltração, 5,6% em diálise peritoneal. Comparado ao ano anterior, houve aumento no uso de cateteres para acesso vascular de hemodiálise, juntamente com maiores taxas de prevalência de anemia, hiperfosfatemia, hiperpotassemia e baixo Kt/V. A taxa bruta estimada de mortalidade anual foi 16,5%.

**Conclusões:**

Dados de uma amostra aleatória de centros de diálise indicam aumento contínuo no número e prevalência de pacientes em diálise crônica no Brasil. Piores tendências em relação ao acesso vascular, adequação da diálise e controle metabólico ressaltam a necessidade de melhorias direcionadas ao cuidado dos pacientes.

## Introdução

Com uma população estimada em 212,6 milhões em julho de 2024, o Brasil ocupa atualmente a 7ª posição entre os países mais populosos do mundo^1^, com uma prevalência de diálise crônica aproximadamente duas vezes superior às estimativas globais^2^.

O custo da terapia renal substitutiva crônica por diálise é financiado pelo governo, por meio do Sistema Único de Saúde (SUS), para a maioria dos brasileiros, há mais de 30 anos. Embora as taxas de reembolso para a terapia dialítica sejam baixas em comparação às de outros países, as taxas de prevalência do tratamento no Brasil são comparáveis às observadas em muitas nações desenvolvidas. A Sociedade Brasileira de Nefrologia (SBN) tem defendido continuamente um reembolso justo por parte do governo. Mais recentemente, empresas internacionais expandiram sua atuação no país como prestadoras de serviços de diálise, contribuindo para uma maior diversidade de opções terapêuticas e uma melhor padronização dos procedimentos.

Há mais de duas décadas, a SBN conduz o Censo Brasileiro de Diálise (CBD), uma pesquisa nacional online desenvolvida para coletar e analisar dados epidemiológicos e clínicos de pacientes em diálise crônica. Essa iniciativa tem sido fundamental para gerar conhecimentos valiosos visando à elaboração de políticas de saúde e ao aprimoramento do cuidado prestado aos indivíduos em tratamento de diálise crônica no Brasil.

Conforme já realizado no CBD de 2023^3^, neste relatório, os dados foram coletados tanto por meio de uma pesquisa voluntária quanto por meio de uma amostra selecionada aleatoriamente de centros de diálise em todo o país. Essa abordagem tem como objetivo aperfeiçoar a precisão das taxas de prevalência e incidência, bem como as estimativas das fontes de financiamento.

O objetivo deste estudo é apresentar os principais achados do CBD 2024 e compará-los aos resultados de pesquisas anteriores.

## Métodos

Trata-se de um estudo transversal, cujo critério de inclusão era se tratar de unidade de diálise com registro ativo na SBN.

### Prevalência, Incidência e Fonte de Financiamento da Diálise

Para estimativas de prevalência, incidência e fonte de financiamento da diálise, os dados foram obtidos por meio de amostragem aleatória a partir de uma lista de centros de diálise de cada região geográfica (Norte, Nordeste, Centro-Oeste, Sudeste e Sul). Os centros foram selecionados por meio de um método de amostragem aleatória computadorizada. Foram selecionados aproximadamente 30% dos centros de diálise de cada região geográfica (n = 258). Foi estabelecida uma meta mínima de 60% (n = 155) do número planejado de unidades de diálise. Essas proporções foram definidas com base em cálculos realizados para o censo de 2023, que gerou estimativas aceitáveis e intervalos de confiança de 95%.

As unidades de diálise selecionadas foram inicialmente contatadas por e-mail, solicitando os seguintes dados: 1. Número total de pacientes em diálise crônica em 1º de julho de 2024; 2. Número de pacientes com planos de saúde privados para financiamento de diálise em 1º de julho de 2024; e 3. Número de pacientes que iniciaram diálise crônica durante julho de 2024. Para aumentar a taxa de resposta, as unidades que não responderam às mensagens eletrônicas foram contatadas por telefone.

Para o cálculo das estimativas, as regiões do país (Norte, Nordeste, Centro-Oeste, Sudeste e Sul) foram consideradas como estratos. Como resultado, foram atribuídos pesos às regiões na fase de análise, a fim de compensar pequenas variações nas probabilidades de amostragem e manter a proporcionalidade (Tabela 1). Considerando que as taxas de resposta foram um pouco diferentes conforme a região, foram atribuídos pesos de modo a permitir que a distribuição da amostra entre os estratos fosse equivalente à observada na população do estudo. Dado o método de amostragem utilizado (amostragem estratificada simples), foi estimado, em 1º de julho, o número médio de pacientes em diálise por centro e calculado o número médio (IC95%) de novos pacientes no ano para cada região. Os resultados foram então expandidos para o número total de unidades em cada região. Os cálculos apresentados na Tabela 1 foram realizados no software Stata utilizando o módulo “svy”, que permite considerar os pesos dos elementos da amostra, a correção para a população finita e a estratificação. As porcentagens de pacientes financiados por planos de saúde públicos e privados foram obtidas por meio de proporção simples na amostra sorteada.

**Tabela 1:** Procedimentos de amostragem em unidades de diálise no país, frações de amostragem, pesos do desenho de pesquisa, taxas de resposta e pesos ajustados por região

Região	Unidades			Fração de amostragem	Peso do desenho	Taxa de resposta	Peso ajustado
	Existentes	Incluídas	Respondentes				
Norte	58	19	14	0,328	3,053	0,737	4,14286
Nordeste	172	48	30	0,279	3,583	0,625	5,73333
Centro-Oeste	91	30	17	0,330	3,033	0,567	5,35294
Sudeste	425	118	72	0,278	3,602	0,610	5,90278
Sul	158	43	29	0,272	3,674	0,674	5,44828
Total	904	258	162			0,628	

Notas – Fração de amostragem = centros incluídos/existentes; Peso do desenho = 1/fração de amostragem; Taxa de resposta = centros respondentes/incluídos; Peso ajustado t = peso do desenho/taxa de resposta.

### Dados da Pesquisa Voluntária

Indivíduos designados pelos centros de diálise participantes preencheram um questionário online disponível no site da SBN, utilizado para coletar dados sobre as características sociodemográficas, clínicas e terapêuticas de pacientes em diálise crônica. O questionário ficou disponível de agosto a novembro de 2024. A participação foi voluntária e todos os centros de diálise registrados na SBN foram convidados por e-mail e pelos canais de mídia da instituição. Para maximizar a participação, lembretes mensais foram enviados aos centros que ainda não haviam submetido os dados, além de ter sido estabelecido contato direto com os presidentes regionais da SBN e com os gestores das prestadoras internacionais de serviços de diálise no Brasil.

Cada centro forneceu dados agregados, em vez de informações individuais dos pacientes, sobre a prevalência de características predefinidas (por exemplo, o número de pacientes em hemodiálise, aqueles com doença renal por diabetes, pacientes do sexo masculino e outros). A mortalidade anual foi estimada com base em ocorrências de julho de 2024. Para o cálculo das taxas de prevalência e incidência, foram utilizados dados populacionais nacionais e regionais de 2024 do Instituto Brasileiro de Geografia e Estatística (IBGE): 212,6 milhões de habitantes^1^.

A maioria dos dados foi descritiva e comparada com informações de anos anteriores. O número anual total estimado de óbitos foi obtido a partir do número de óbitos relatados em julho, multiplicado por 12 e dividido pela proporção de centros participantes ativos. A taxa bruta de mortalidade anual estimada (%) foi obtida a partir do número total estimado de óbitos em 2024, multiplicado por 100 e dividido pelo número estimado de pacientes em diálise em 1º de julho na amostra voluntária.

Este estudo não envolveu acesso a dados individuais primários ou secundários de pacientes. Todas as informações foram obtidas de forma agregada, conforme relatado pelos centros de diálise participantes por meio de um questionário online padronizado.

## RESULTADOS

Em julho de 2024, um total de 904 centros ativos de diálise crônica estavam registrados na SBN, representando um aumento de 2% em relação a 2023. Em âmbito nacional, havia 4,2 centros de diálise por milhão da população (pmp). Foram observadas disparidades regionais, com taxas mais baixas no Nordeste (3,0 pmp) e Norte (3,1 pmp) em comparação a taxas mais elevadas nas regiões Sudeste (4,8 pmp), Centro-Oeste (5,3 pmp) e Sul (5,1 pmp).

### Estimativas da Amostra Aleatória

#### Taxas Estimadas de Incidência e Prevalência a Partir da Amostra Aleatória

Dezoito por cento dos centros de diálise ativos no Brasil participaram da amostra aleatória (n = 162/ 904), 24% da região Norte (n = 14/58), 17% do Nordeste (n = 30/172), 19% do Centro-Oeste (n = 17/91), 17% do Sudeste (n = 72/425) e 18% do Sul (n = 29/158). Os centros representaram 62% do total planejado (162 de 258).

Em 1º de julho de 2024, o número total estimado de pacientes em diálise foi de 172.585 (IC95%: 156.687–188.483), refletindo um aumento de 9,7% em relação a julho de 2023—o maior aumento anual observado nos últimos anos (Figura 1). A prevalência de pacientes em diálise também continuou a aumentar, de 771 pmp em 2023 para 812 pmp (IC95%: 737–887 pmp) em 2024. Regionalmente, a taxa de prevalência apresentou redução no Nordeste (Figura 2).

**Figura 1.** Estimativa do número de pacientes em diálise crônica por ano.

**Figura 2.** Taxa estimada de prevalência de pacientes em diálise por região geográfica no Brasil, por milhão da população.

O número estimado de novos pacientes em diálise em 2024 foi de 52.944, com uma taxa de incidência global de 249 pmp (IC95%: 220–279 pmp), comparável à taxa de 2023, de 251 pmp. As taxas de incidência variaram entre as regiões, oscilando entre 120 pmp no Norte e 311 pmp no Sudeste.

Com base na pesquisa por amostra aleatória, 79% dos pacientes em diálise eram financiados pelo sistema público de saúde, enquanto 21% dependiam de planos de saúde privados. O financiamento público variou por região, sendo mais elevado no Norte (91%), seguido pelo Nordeste (80%), Sudeste (78%), Sul (76%) e Centro-Oeste (70%).

### Pesquisa Voluntária

Um total de 386 centros de diálise (43%) participou da pesquisa voluntária, a maior taxa de resposta registrada desde 2014. Ao analisar a participação por região, o Nordeste apresentou a maior taxa de resposta (49%), seguido pelo Sul (46%), Centro-Oeste (42%), Sudeste (40%) e Norte (34%).

O número de pacientes incluídos no atual CBD foi 22,9% superior ao de 2023, aumentando de 62.617 para 76.946. O número estimado de óbitos para o ano foi de 29.712, resultando em uma taxa bruta de mortalidade anual de 16,5% (Figura 3).

**Figura 3.** Taxa estimada de mortalidade bruta anual de pacientes em diálise.

#### Características Demográficas e Clínicas

Cinquenta e nove por cento dos pacientes em diálise eram do sexo masculino e 41% do sexo feminino. Quanto à distribuição etária, 0,8% tinham menos de 20 anos, 61,4% tinham entre 20 e 64 anos e 37,8% tinham 65 anos ou mais.

A hipertensão e o diabetes permaneceram como as principais causas primárias da doença renal, cada uma representando 29% dos casos (Figura 4). A prevalência de pacientes com sorologia positiva para hepatite C, hepatite B e HIV permaneceu estável e semelhante à de 2023 (Figura 5).

**Figura 4.** Distribuição dos pacientes em diálise de acordo com a etiologia da doença renal crônica.

**Figura 5.** Prevalência de pacientes com sorologia positiva para hepatite B e C e HIV.

Em relação ao acesso vascular para hemodiálise ou hemodiafiltração, cerca de um quarto dos pacientes (23%) utilizava um cateter de longa permanência, enquanto a prevalência de fístula arteriovenosa apresentou redução (Figura 6).

**Figura 6.** Tipos de acessos vasculares utilizados para hemodiálise.

A maioria dos parâmetros bioquímicos apresentou piora em comparação ao ano anterior, particularmente a prevalência de potássio sérico ≥ 6,0 mEq/L, que aumentou de 13% para 19%, e de Kt/V inadequado, que saltou de 21% para 24%. Além disso, 21% dos pacientes apresentaram paratormônio >600 pg/mL, 31% apresentaram níveis de hemoglobina <10 g/dL e um terço da população apresentou fosfato sérico >5,5 mg/dL (Figura 7).

**Figura 7.** Distribuição dos pacientes em diálise de acordo com os resultados bioquímicos.

As principais alterações no uso de medicamentos em comparação ao ano anterior foram a redução no uso de eritropoietina (74% para 70%), ferro intravenoso (51% para 46%) e calcitriol (19% para 12%) (Figura 8).

**Figura 8.** Distribuição dos pacientes em diálise de acordo com o uso de medicamentos.

#### Características do Tratamento Dialítico

A distribuição das modalidades de diálise e fontes de financiamento na pesquisa voluntária é apresentada na Tabela 2. O sistema público de saúde financiou 80,7% dos pacientes (n = 62.084/76.946), enquanto os planos de saúde privados cobriram 19,3% (n = 14.862/76.946).

**Tabela 2:** Distribuição dos pacientes por modalidade de diálise e fonte de financiamento

Modalidade	Saúde pública		Saúde privada		Total	
	N	%	N	%	N	%
HD ≤ 4 sessões/semana	58734	94,6	8006	53,6	66740	86,7
HD > 4 sessões/semana	0	0	411	2,8	411	0,5
HD Domiciliar	0	0	29	0,2	29	0,0
HDF ≤ 4 sessões/semana	201	0,3	4560	30,7	4761	6,2
HDF > 4 sessões/semana	0	0	663	4,5	663	0,9
HDF Domiciliar	0	0	5	0	5	0
DPAC	308	0,5	127	0,9	435	0,6
DPA	2771	4,5	1029	6,9	3800	4,9
DPI	70	0,1	32	0,2	102	0,1
Total	62084	100	14862	100	76946	100

Abreviaturas – HD: hemodiálise; HDF: hemodiafiltração; DPAC: diálise peritoneal ambulatorial contínua; DPA: diálise peritoneal automatizada; DPI: diálise peritoneal intermitente.

A proporção de pacientes submetidos à hemodiálise e hemodiafiltração apresentou uma ligeira redução, passando de 88,2% para 87,3% e de 8,0% para 7,1%, respectivamente. Em contrapartida, o uso de diálise peritoneal aumentou em nível nacional, de 3,8% para 5,6% (Figura 9), e em todas as regiões, exceto no Norte.

**Figura 9.** Distribuição dos pacientes em diálise de acordo com a modalidade de diálise.

Mais de 10% dos pacientes em hemodiafiltração realizavam ao menos cinco sessões por semana, enquanto apenas uma minoria de pacientes em diálise peritoneal não utilizava a modalidade automatizada (Figura 10).

**Figura 10.** Distribuição dos pacientes de acordo com a prescrição de diálise.

Em relação às características do dialisato, 60% das unidades de diálise utilizavam a mesma concentração de bicarbonato para todos os pacientes e 62% utilizavam a mesma concentração de potássio.

#### Características dos Centros Participantes

Entre os 386 centros de diálise participantes, 76% eram privados, 15% eram filantrópicos e 9% eram públicos. Além disso, 27% dos centros eram operados por empresas internacionais.

A maioria dos centros foi classificada como unidades satélites (68%), enquanto 32% eram centros hospitalares. A média nacional de pacientes por nefrologista, enfermeiro e técnico de enfermagem foi de 25, 41 e 7, respectivamente.

## Discussão

Neste estudo, apresentamos os principais achados da edição de 2024 do CBD. Este ano registrou a maior taxa de participação da última década, refletindo um maior engajamento com a pesquisa. A tendência mais significativa observada foi um aumento na prevalência de parâmetros bioquímicos inadequados, ressaltando os desafios contínuos para alcançar a adequação do tratamento e o controle metabólico eficaz.

Presumimos que o aumento expressivo no número estimado de pacientes em diálise, de quase 10% em um ano, possa refletir principalmente dois fatores: o envelhecimento da população brasileira em razão de uma maior expectativa de vida^4^ e as taxas de mortalidade mais baixas observadas após os anos da pandemia de COVID-19. No entanto, a precisão dessa estimativa, baseada em dados amostrais, deve ser confirmada em pesquisas futuras.

A prevalência de diálise aumentou de 771 para 812 pmp, enquanto as disparidades regionais persistiram, provavelmente refletindo as desigualdades no acesso ao tratamento em todo o país. Em média, a taxa de prevalência manteve-se 35% mais elevada nas três regiões mais desenvolvidas – Centro-Oeste (887 pmp), Sudeste (1.019 pmp) e Sul (708 pmp)—em comparação às regiões menos desenvolvidas, como Norte (567 pmp) e Nordeste (608 pmp), com uma média de 902 pmp contra 587 pmp, respectivamente. Essas discrepâncias ressaltam a influência de fatores socioeconômicos e de infraestrutura no acesso à terapia renal substitutiva. Uma pesquisa recente, abrangendo dados de 167 países, relatou variação substancial na prevalência de diálise crônica, com mediana de 322,7 pmp para hemodiálise (intervalo interquartil [IIQ] 76,3–648,8)^2^ e 21,0 pmp para diálise peritoneal (IIQ 1,5–62,4)^5^. Esses dados contextualizam a posição do Brasil como um país com cobertura de diálise relativamente elevada, comparável à de várias nações desenvolvidas, embora ainda marcado por desequilíbrios internos significativos.

Observa-se uma disparidade ainda maior na taxa de incidência, que é 42% mais elevada nas regiões mais desenvolvidas (273 pmp) em comparação às regiões menos desenvolvidas (156 pmp). Isso evidencia as desigualdades persistentes não apenas no tratamento em curso, mas também no início da diálise. Em nível nacional, a taxa de incidência permaneceu relativamente estável, com uma ligeira redução de 251 para 249 pmp em comparação ao ano anterior.

Curiosamente, pela primeira vez desde o primeiro CBD, o diabetes—tradicionalmente a segunda causa principal—igualou-se à hipertensão como causa primária de doença renal, ocupando conjuntamente a primeira posição. Concomitantemente, houve um aumento na prevalência de causas primárias classificadas como outras ou desconhecidas, que, em conjunto, representaram quase um terço dos casos (31%). Em outros registros internacionais, como o USRDS dos Estados Unidos e o Registro Europeu, o diabetes tem sido consistentemente a principal causa de falência renal na última década^6,7^. A recente convergência entre diabetes e hipertensão como causas primárias em nossos dados reflete uma tendência semelhante e reforça a necessidade de estratégias preventivas focadas na doença renal relacionada ao diabetes.

No que diz respeito à sorologia positiva, as leves flutuações observadas nos últimos anos para o HIV e a hepatite B persistiram. Notavelmente, a prevalência de hepatite C não apresentou redução pela primeira vez desde 1999, mantendo a prevalência de 2,1% registrada em 2023. A vigilância contínua e o reforço das práticas de triagem e controle de infecções permanecem fundamentais nas populações em diálise.

O uso de fístulas arteriovenosas para acesso em hemodiálise manteve-se em declínio, enquanto os cateteres de longa permanência passaram a constituir o acesso vascular de quase um quarto dos pacientes. Essa tendência pode, em parte, refletir o aumento da expertise dos nefrologistas na inserção de cateteres de longa permanência, aumentando sua disponibilidade e percepção de conveniência, especialmente quando comparados aos cateteres não tunelizados. No entanto, também levanta preocupações sobre o potencial comprometimento dos desfechos de longo prazo, dada a menor permeabilidade e os riscos mais elevados de infecção, hospitalização e mortalidade associados ao uso de cateteres em comparação às fístulas arteriovenosas^8,9^. Esses achados ressaltam a necessidade de equilibrar a conveniência com a adesão às melhores práticas no planejamento do acesso vascular, particularmente o encaminhamento oportuno para avaliação cirúrgica e a educação do paciente.

Em comparação a 2023, a prevalência de parâmetros bioquímicos inadequados aumentou em quase todos os indicadores, destacando potenciais desafios na adequação do tratamento e no controle metabólico. A proporção de pacientes com Kt/V < 1,2 aumentou de 21% para 24%, sugerindo um declínio na administração da dose de diálise ou na adesão ao tratamento. De forma semelhante, a taxa de hipercalemia aumentou de 13% para 19% e a de paratormônio elevado de 19% para 21%. Enquanto isso, as taxas de hiperfosfatemia permaneceram persistentemente elevadas, afetando aproximadamente um terço dos pacientes, o que indica dificuldades contínuas em alcançar o controle do fósforo, apesar das opções terapêuticas disponíveis. Recentemente, em uma pesquisa nacional envolvendo 114 centros de diálise, aproximadamente 30% relataram dificuldades em prescrever pelo menos 12 horas de hemodiálise por semana^10^, o que pode explicar parcialmente esses achados. A mesma pesquisa também constatou que quase 20% dos pacientes apresentam hiperparatireoidismo grave e que muitos serviços possuem acesso limitado tanto ao tratamento clínico quanto ao cirúrgico para essa condição^10^.

A prevalência da anemia manteve-se elevada, afetando 31% dos pacientes, enquanto o uso dos principais tratamentos – eritropoietina e ferro intravenoso – atingiu seus níveis mais baixos desde o início da coleta de dados para essas variáveis em 2008, com 70% e 46%, respectivamente. A redução no uso terapêutico, apesar das taxas persistentes de anemia, é preocupante, e as razões por trás dessas tendências merecem uma investigação mais aprofundada, especialmente considerando a associação bem estabelecida entre anemia não tratada e piores desfechos em pacientes em diálise^11^.

Conforme observado no ano anterior, houve um declínio contínuo no uso de calcitriol, juntamente com um aumento no uso de cinacalcete e paricalcitol. Essa mudança provavelmente reflete a evolução de estratégias clínicas destinadas ao manejo mais eficaz do hiperparatireoidismo secundário, minimizando o risco de hipercalcemia e calcificação vascular^12^. Além disso, fatores externos podem ter contribuído para essas tendências, como a descontinuação do calcitriol intravenoso no mercado. Uma pesquisa nacional anterior também constatou que uma proporção significativa das unidades de diálise relatou dificuldades na obtenção de medicamentos relacionados ao DMO-DRC, o que pode influenciar ainda mais os padrões de tratamento^10^.

O uso da diálise peritoneal aumentou de 3,8% para 5,6%, revertendo o declínio observado nos últimos dois anos. Em contraste, o uso da hemodiafiltração apresentou uma modesta redução de 8% para 7%, e o da hemodiálise, de 88% para 87%. Entre as possíveis explicações para a retomada da diálise peritoneal estão a maior taxa de participação na presente pesquisa e o impacto de políticas públicas em diversos estados voltadas à promoção dessa modalidade.

Entre as limitações do estudo, destacam-se a dependência da coleta eletrônica de dados por meio de participação voluntária, a agregação dos dados de pacientes em nível de centro de diálise e a falta de validação das respostas. Além disso, uma vez que as taxas de incidência e prevalência foram calculadas utilizando métodos de amostragem diferentes em relação aos anos anteriores (antes de 2023), as comparações ao longo do tempo devem ser interpretadas com cautela. Como ponto forte, o uso de um método de amostragem aleatória para a seleção de centros de diálise em nosso extenso país, que conta com mais de 900 instalações ativas, aumentou a validade das estimativas de prevalência e incidência, bem como a precisão das distribuições dos tipos de financiamento da diálise.

Em conclusão, o CBD 2024 confirmou o aumento contínuo da prevalência de pacientes em diálise no Brasil, juntamente com tendências preocupantes no acesso vascular, incluindo uma redução no uso de fístulas arteriovenosas. Além disso, a elevada proporção de pacientes com doses inadequadas de diálise e controle metabólico deficiente destaca a necessidade de intervenções direcionadas para aprimorar os cuidados de diálise e os desfechos dos pacientes.

## Data Availability

Os dados que embasam os achados deste estudo estão disponíveis na Sociedade Brasileira de Nefrologia (SBN), mas há restrições aplicáveis. Os dados não estão disponíveis publicamente devido a considerações éticas e de privacidade, mas podem ser obtidos junto aos autores mediante solicitação razoável e com a permissão da SBN.

## Material Suplementar

O seguinte material online está disponível para o presente artigo:

Questionário do CBD 2024.
